# Labneh: A Retail Market Analysis and Selected Product Characterization

**DOI:** 10.3390/foods13213461

**Published:** 2024-10-29

**Authors:** Raman K. Bhaskaracharya, Fatima Saeed Rashed Alnuaimi, Shaikha Rashed Juma Aldarmaki, Abeena Abdulazeez, Mutamed Ayyash

**Affiliations:** Department of Food Science, College of Agriculture and Veterinary Medicine, United Arab Emirates University, Al Ain P.O. Box 15551, United Arab Emirates

**Keywords:** extended shelf life, formulation, labneh, rheology, texture

## Abstract

Labneh is a popular fermented dairy product, which contemporarily has diversified into a varied range of styles, formulated with the inclusion of multiple additives, and is sourced across the globe. This has driven labneh’s emergence as a complex product with varying textural and rheological characteristics. The lack of scientific literature about labneh products available in the United Arab Emirates (UAE) market and their characterization has prompted this study. A detailed UAE market analysis of labneh for label, formulation, nutrition, and price variability was conducted. Surveyed labneh products were categorized as unpackaged, multinational company (MNC), small and medium enterprise (SME), and specialty products. They differed in manufacturing, such as acid ± enzyme coagulation with/without post-fermentation heat treatment, and contained various stabilizers, emulsifiers, preservatives, and processing aids. Interestingly, almost equal proportions, 64.7% and 67%, of MNC and SME labneh contained additives, respectively. All MNC labneh were post-heat-treated, in contrast to only 7% of SME labneh. Organic labneh and non-bovine milk-based labneh are not yet widely available. The second part of the study involved the physicochemical characterization of a select number of packaged labneh that were categorized in accordance with fat content as high-fat (17–18%), full-fat (7.1–8%), and lite-fat (3.5–4.5%). High-fat labneh showed a significantly higher complex viscosity, complex modulus, hardness, adhesiveness, stringiness, and fracturability, followed by lite-fat labneh compared to full-fat labneh, especially when it contained pectin. Full-fat labneh with added gums (and starch) and high-fat labneh with gums showed a significantly lower complex modulus compared to their respective control labneh. This study highlights the variety of commercial labneh products available and differences in their formulation, manufacturing, and composition, and provides specific dependencies of materials with their physicochemical characteristics.

## 1. Introduction

Labneh is a coagulated milk product obtained by fermentation of milk or milk products using starter cultures, namely lactic acid bacteria. The post-fermentation consistency is thickened by reducing the water content [1]. There are several classifications of labneh according to the GCC standard [1], including regular labneh, labneh-in-oil, labneh heat-treated after fermentation, and Khazine labneh (more solid in consistency than regular labneh and packaged in suitable containers). Labneh products are popular in North African, Middle Eastern, and Gulf Cooperation Council (GCC) countries [2,3] and are known in European countries with different names but similar characteristics [3]. Regular labneh is traditionally produced by prolonged fermentation of bovine milk using lactic acid bacteria such as *Lactobacillus bulgaricus* and *Streptococcus thermophilus*. Prolonged milk fermentation using starter culture and or native microflora produces intense flavor compounds via proteolysis and lipolysis pathways. Afterward, the fermented milk is salted to ~1.5% [4] and filtered using cheesecloths (bag straining) overnight at room temperature under gravity or with externally applied pressure [5]. The labneh usually has an acidity of 1.2–2.5% lactic acid [1] and the moisture content varies depending on the type of labneh [6]. Hard labneh-type products possess a low moisture content of ~45–50% and an acidic taste (pH~4.3) with a high viscosity [7]. The fresh labneh typically has a characteristic texture, slightly acidic taste, and lactic acid odor [6].

Industrially, labneh is produced using centrifugation techniques to reduce the moisture content after milk fermentation [8]. Other techniques for commercial manufacturing of labneh use ultrafiltration techniques to concentrate milk before fermentation [8]. The resulting product is characterized as a low viscosity labneh product. Furthermore, both commercial manufacturing methods produce low acid labneh-type products due to the short fermentation time.

Several attributes, such as taste, moisture content, homogeneity, and viscosity, are the main determinants of labneh’s quality [3]. Syneresis leading to whey separation is a significant defect found in labneh’s final product. Usually, the shelf life of the traditionally made labneh is between 10 and 15 days at 4 °C [6]. The short shelf life of the labneh has been reported as one of the limitations of its commercial production. It has been reported that for packaged labneh under refrigerated storage, psychotropic yeasts are the main cause of spoilage. Therefore, inclusion of ‘generally recognized as safe’ (GRAS) status food preservatives such as sorbic and benzoic acids has been cited and recommended [9]. The use of food-grade preservatives (e.g., sorbate) has been employed commercially as a strategy to control fungal growth during storage and maintain the shelf life of the labneh [10].

Post-fermentation acidification during storage is also one of the factors that influence the labneh’s shelf life and its quality attributes. The physicochemical changes, such as syneresis of whey and high acidity, and microbiological changes, including decreased LAB counts, gas production, and undesired flavor changes by yeast or coliform contaminants, affect acceptability of labneh [11]. The logistics of cold chain control are often inefficient in controlling post-acidification in fermented dairy products, especially in the tropical climates, thus reducing the shelf life [11]. Post-fermentation heat treatment has been suggested as an effective strategy to prevent post-fermentation acidification [12]. The heat treatment applied to labneh after fermentation is primarily to kill the initial starter cultures and any adventitious microorganisms. This is expected to stop the bacteria from producing more lactic acid and prevent excess production of metabolic compounds that can impart undesirable flavors in the labneh [11]. The heat treatment also helps to provide a pasteurization treatment, thereby giving a slightly cooked flavor [13].

Fat-derived flavor compounds, including volatile fatty acids, esters, aldehydes, ketones, lactones, alcohols and non-volatile long chain fatty acids, formed during fat reduction provide flavor to labneh. Fat also plays a crucial role in labneh texture and other physical characteristics such as consistency and opacity. However, the health concerns related to fat-rich products have driven the recent consumer trends towards a preference for low-fat labneh [14]. Based on its fat and non-fat solid (SNF) contents, labneh can be classified as full-fat labneh, low-fat labneh and skimmed fat labneh. These categories of labneh are expected to have differences in their physicochemical characteristics [14]. Hydrocolloid additions as a fat substitute can enhance the texture and sensory attributes of reduced-fat labneh to a similar profile as that of a full-fat Labneh [15].

Food-grade hydrocolloids are commonly used in industrial manufacturing of high-moisture labneh to prevent/control syneresis by improving the water-holding capacity of the final product [15,16]. The hydrocolloids are typically added during the milk processing prior to the fermentation step [15]. Commercial labneh therefore includes stabilizers and preservatives to bring the consistency of their products close to traditional labneh. GCC standards allow for additives and processing aids to be used, as per Codex Alimentarius [1].

The professed health benefits of labneh have led to its popularity, positioning it as one of the bestselling dairy products all over the world [14]. However, in recent times, the perceived negative connotations due to formulation changes that include food additives and preservatives have driven customer demand for clean label food products. Clean labels are a consumer term (non-scientific), but widely accepted by food manufacturers, the scientific community, and regulatory agencies. Clean labels represent consumers’ idea of products with fewer ingredients, especially those that the consumers can recognize as wholesome, and not artificial ingredients or synthetic compounds. This desire by consumers for natural and clean label food products has in turn triggered the efforts of food companies to reformulate their products to minimize complicated formulations with too many additives. Moreover, the use of excessive ingredients and additives adds to the cost of products, thereby reducing profitability. Current research suggests that from the consumers’ perspective, foods with shorter/minimal processing involving fewer and specialized/highly functional protein ingredients that could partially replace additives would be preferred by consumers seeking “clean label” foods [17,18]. In recent times there has been an increasing consumer demand for nutritious, “reduced fat” clean label dairy products. Consumer acceptability is the final goal, and despite consumer awareness of healthier “clean label” products, a similarity in taste, texture, and aroma to the conventional products is still expected by consumers [19]. However, ingredient list modification to adapt to the “clean label” consumer requirement pose a challenge to the manufacturers [20].

Differences in formulation design and processing conditions can cause variations in textural and rheological characteristics of labneh. The quality attributes can also vary significantly between manufacturers of the same type of labneh [21]. A recent study of fermented dairy products reported that the scale of Greek-style yogurt production and type of process affected its sensory and physicochemical characteristics [22]. Furthermore, the impact of the method of production, i.e., large (industrial) scale or small scale was reported to have a significant effect on the acceptability of the texture, flavor, and appearance of labneh [21].

There is a lack of currently available information on nutritional, label, formulation, processing variability, and physicochemical comparisons of labneh products in the UAE and the world over. Hence, a detailed analysis of the market situation is warranted. This study evaluated all available information on labneh products from the current consumer perspective. The impact of formulation, including various additives, and differences in processing conditions on the labneh products’ texture and rheology also were explored. These differences are the focus of this study, especially as seen in labneh sold in local UAE markets (sourced from GCC countries) from which major labneh products are imported. Specifically, this study aimed to assess commercial packaged labneh sold locally in the UAE, compare their quality attributes, investigate their chemical composition, study differences in their formulation, and evaluate their rheological and textural properties, highlighting the type of hydrocolloids used in these products. The study focused on regular labneh, labneh heat-treated/not post-fermentation, and khazine labneh that are sold in a packaged format. The unpackaged (loosely sold) labneh and labneh in oil were only considered for preliminary oversight.

## 2. Material and Methods

*Part 1. Retail Market Analysis*

The study was conducted in two parts, and for the first part, a survey of the labneh products was conducted across UAE by accessing product information from dairy and food online retailers and through personal visits to investigate at several local retail stores. A total of 13 retail chains/stores were surveyed. An in-depth review of labneh products was undertaken to collate the product descriptions (from the retailer website/in store), list of ingredients (from the label, including preservatives, emulsifiers, stabilizers, added vitamins, salt, etc.), declared nutritional profile as evidenced from the product label, price (converted to AED per 100 gm), and country of origin (from label). Since limited product information and compositional data was available for the labneh that was sold loose, the search was focused on Labneh sold in a packaged format.

*Part 2. Selection of Packaged Labneh Products for Physicochemical Analysis*

The second part of the project involved an in-lab investigation of purchased labneh available in the UAE. Stratified according to the fat content (the fat content of collected samples was above 3.5%), the samples were classified as high-fat (17–18% fat), full-fat (7.1–8% fat) and lite-fat (3.5–4.5% fat). Based on the formulation, processing, and compositional differences, a total of 8 packaged labneh (3 labneh containing high-fat, 3 labneh containing full-fat, and 2 labneh containing lite-fat levels) were selected to study the relationship of the formulation and processing to their rheology and texture characteristics. Three samples of each of the selected labneh were purchased with same expiry or best before date and coded as 3 high-fat (creamy) labneh category samples—H1, H2, and H3; 3 full-fat labneh category samples—F1, F2, and F3; and 2 lite-fat labneh category samples—L1 and L2. A third lite-fat category labneh was unavailable during the study, hence only two lite-fat labneh packaged products could be included. Each sample was analyzed in triplicate.

### 2.1. Rheological Analysis

Dynamic rheological measurements were carried out employing a previously published methodology with slight modifications [23]. Briefly, rheological tests were performed using a Discovery Hybrid Rheometer HR-2 (TA Instruments, Wilmington, DE, USA) attached to a 40 mm parallel plate geometry (Peltier plate Steel). After loading, samples were allowed to relax and equilibrate to 25 °C (2 min) prior to running the test with the gap maintained constant at 1.0 mm throughout all the tests. The oscillation frequency sweep was carried out from a high to low frequency (range 0.02–15 Hz) at 0.119 mNm torque and the oscillation amplitude sweep with a torque range of 0.01–3 mNm at 0.25 Hz frequency was performed. A water circulator ensured maintenance of a constant temperature of the sample loaded on the rheometer. A minimum triplicate sample analysis was performed for the frequency sweep and for the amplitude sweep measurements on at least two separate days with at least ten readings taken from each replicate to measure the dynamic parameters of the storage modulus (G′) and loss modulus (G″). The standard errors were less than ±8% of the average values for each sample. The yield point, also called the linearity limit, was determined for each sample as the value of the shear stress taken at the limit of the LVE region. Similarly, the flow point for each sample was determined as the value of the shear stress at the crossover point of G′ and G″. The flow transition index was calculated as shown in the formula below:

(1) $$Flow\;Transition\;Index=\frac{Flow\;Point}{\mathrm{Yield}\;\mathrm{Point}}$$

### 2.2. Texture Analysis

Texture analysis was carried out in triplicate using a TexturePro CT V1.3 Build 15 (Brookfield AMETEK, Middleboro, MA, USA) texture analyzer controlled by a computer. Samples were analyzed immediately after being taken out of the refrigerator. The texture analyzer was fitted with a rotary base table, TA-RT-KI, for all sample analysis. A normal test of a single compression cycle was achieved using a TA5 black delrin 5 g cylindrical probe measuring 12.7 mm in diameter, 35 mm in length, and 0.35–0.43 mm in rad. A 1500 g load cell was used for compression tests. The probe was set to travel at a 0.5 mm/s speed to a target distance of 15 mm during both compression and retraction according to a previously reported method [23]. The Texture Pro CT (Brookfield AMETEK, MA, USA) was used to set up and collect data at 10 points/s from the force measurements to calculate hardness, adhesiveness, cohesiveness, chewiness, etc.

### 2.3. Statistical Analysis

The results obtained from the experiments in triplicate for each analysis were subjected to statistical analyses using GraphPad Prism version 9.3.0 (GraphPad Software, LLC, California, USA). Proximity analysis was evaluated using Tukey’s multiple comparison test with one-way ANOVA. The nutritional parameters were compared between the MNC and SME products using Mann–Whitney *t*-test. The median and 75% interquartile range was indicated by a dashed line and two dotted lines in the density plot of the figures. All texture results were analyzed with two-way ANOVA using Tukey’s multiple comparison test. Confidence intervals for 95% confidence and specific statistical significance were calculated using an alpha of 0.05.

To characterize the multidimensional data of texture, rheology, and composition, and to summarize their individual within- and between-group variations, Principal component analysis (PCA) was performed. The texture attributes, such as hardness, stringiness, fracturability, and adhesiveness, were included along with the rheological measurements of tan δ, yield point, flow point, flow transition index, complex modulus, complex viscosity etc., which were analyzed together with the compositional characteristics of protein and fat % for primary analysis. A secondary PCA analysis of textural characteristics with composition parameters was also undertaken. All statistical calculations and graphs were prepared using GraphPad Prism version 9.3.0.

## 3. Results and Discussion

As stated earlier, this study was carried out in two parts. The first part involved carrying out a market analysis to identify and collect information on all the labneh retail products in the UAE market. Labneh product availability across UAE was evaluated by accessing online stores of dairy and food retailers and in-store investigations at retail stores. A total of 13 retail chains/stores were surveyed and 116 labneh products identified. Part 1 provides the descriptors used by retailers outlining the products, ingredients listed on the package, the price, and the reported nutritional content on the label. The company websites were also used to collect some of the labneh label-related information if not available on the pack/label. The second part of the study entailed evaluating the composition, rheology, and textural characteristics of the selected eight labneh products retailed in the UAE market, while examining if differences in formulation, ingredients, and/or processing conditions impacted their physicochemical characteristics.

*Part 1: Retail Market Analysis*

### 3.1. Country of Origin & Labneh Product Descriptors

**Figure 1:**
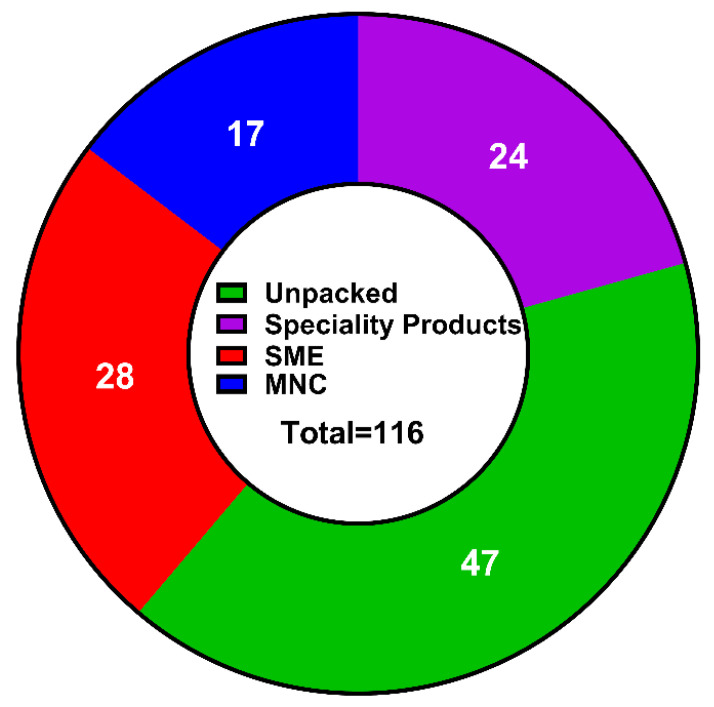
Market analysis of commercial labneh in UAE market.

Among the identified 116 labneh products, 47 products were sold loose (unpackaged/bulk sales) of which 42 products were manufactured locally, while 5 products were imported into the UAE market. The remaining 69 labneh were sold as packaged, of which 28 were manufactured by small and medium enterprises (SMEs, 23 are manufactured locally, and 5 are imported), 17 packaged labneh products were manufactured by multinational companies (MNCs, only 2 made locally). Additionally, 24 (out of 69) packaged labneh were specialty products sold in retail stores, all manufactured locally (Figure 1).

Increased awareness and perception have led to an increased demand for local foods by consumers [24]. Interestingly, according to the label declaration, the majority of labneh products included in our study were manufactured locally; however, in contrast, the majority of the MNC products were imported, with only a few products listed as manufactured locally.

The product description of the unpackaged labneh on the retailers’ website and/or product stickers in-store included terms such as “Authentic”, “Fresh”, “Soft”, “Deli Selection”, “Hard”, “Labaneh Jarashieh”, “Full-fat”, and “Low Fat” labneh or labneh ball, and are also described based on herbs/spices/condiments used. The products manufactured by SMEs and MNCs were described on labels with terms such as “Lactose free”, “Fresh”, “Cheese Spread with extra labneh taste”, “Full-fat”, “Thick and Creamy”, “Full Cream”, “Premium”, “Organic”, “Low Fat/light/lite”, “Jarshia”, “Fresh”, and “Deli Selection”. The terms used to describe products on the label of specialty products included “Herbed labneh”, “Plain Labana”, “Labana Salad”, “Plain Labana”, “Spheral-sheep”, “Spheral”, and descriptors based on herb/non-dairy ingredient used (Table 1).

### 3.2. Style of Product, Availability in Retail, and Economic Dimension

The unpackaged labneh products retailing in the UAE market were available as “labneh”, “labneh ball” and “labneh analogue” in plain and herbed format and were labeled as “Turkish”-and “Labanese”-style products. The packaged labneh manufactured by MNCs and SMEs were available in various formats, such as “labneh ball”, “labneh”, “Original—(in creamy, full-fat, low fat formats)”, “lactose free”, “Organic”, “Turkish Style”, “Hungarian Style” and “Lebanese Style”. The products retailing in the UAE market in the format of a “labneh mix” were categorized as a specialty product (Table 1).

Unpackaged labneh products were retailed at stores such as Carrefour, Lulu supermarket, Spar supermarket, Spinneys, Choitrams, and Grand Hyper Mart. The SME and MNC manufactured labneh products were sold at various retail chains/stores, including Lulu supermarkets, Carrefour, Grand Hyper Mart, Minbaladeh, Al Ain Co-op, Sharjah Co-op, Spar Supermarket, Spinneys, Choitrams, Noon, Desert Cart, Amazon.ae, and Gradoise. In contrast, the specialty products were sold only in limited stores such as Lulu, Amazon.ae, and Sharjah Co-op (Table 1).

Unpackaged labneh products retailed for 100 g at a price point of United Arab Emirates Dirham (AED) 3.91 ± 0.1. The retail price point of the SME and MNC packaged labneh products were similarly priced at AED 3.551 ± 0.41 and AED 4.344 ± 0.38 AED/100 g, respectively. The specialty products, on the other hand, retailed at a premium price of AED 5.875 ± 0.22 AED/100 g, which was significantly higher than that of the labneh products sold loose (*p* < 0.001) or manufactured by MNCs (*p* < 0.05) and SMEs (*p* < 0.001).

### 3.3. Ingredient List per Label Declaration

Labneh sold as unpackaged were excluded from further analysis due to limited product and compositional data availability for these products. The ingredients listed on the products manufactured by the MNCs ranged between 3 and 21 ingredients, while the SME products had 3 to 9 ingredients listed on their labels. The label of the specialty products, however, lacked clarity because either the ingredients were not listed and/or the product ingredients included labneh as one of the ingredients. The other herbs/spices/condiments included in the specialty products were listed on the ingredient label in addition to labneh as an ingredient. The limited ingredient information of the specialty products therefore precluded them from further analysis.

The labneh products manufactured by the SMEs and MNCs were next compared to assess if the manufacturer’s size of operation resulted in differences in product characteristics.

### 3.4. Product Characteristics of SME vs. MNC Labneh

Differences in the microbial, physicochemical, and sensory properties between labneh samples produced at a large scale (branded) versus a small scale (unbranded) has been previously reported in the literature [25]. However, a comparison of the labneh product characteristics based on the scale of the manufacturing has not been evaluated. The current study therefore examined if the scale of manufacturing i.e., SMEs versus MNCs, impacted the product diversity and product characteristics of the labneh available in the UAE market.

**Figure 2:**
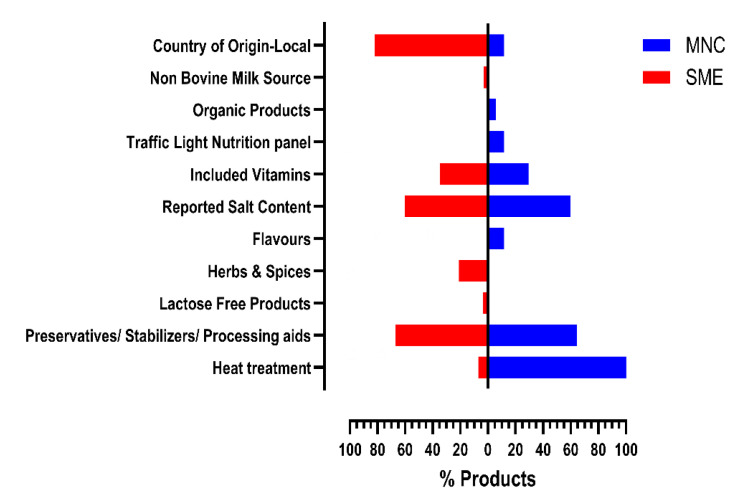
Product characteristics of SME vs. MNC labneh.

Although none of the MNC labneh products available in the UAE retail market used a non-bovine milk source, only one labneh product (3.57%) among the SME products surveyed used non-bovine milk as the milk source (Figure 2). It has been proposed that the non-bovine milks have unique properties that offer technological advantages in food manufacturing, contribute to the product diversity, and caters to the variations in consumer preferences for these products [26].

The surveyed products that catered to specific consumer requirements such as organic products or lactose-free products were also evaluated. Among the surveyed labneh products available in the UAE market, one (5.88%) MNC labneh product was organic, while none of the surveyed SME labneh products were organic (Figure 2). In contrast, among the 116 labneh products surveyed, only one (3.57%) was available as a lactose-free product that was manufactured by an SME among the MNC products (Figure 2).

Nutrition details on labels are employed to convey nutrition information to consumers. However, a move to a nutrition facts panel such as traffic light labeling can effectively convey information about the perceived healthiness of the food item [27]. The government of UAE recently launched a National Nutrition Strategy, wherein some of the healthy food environment measures were introduced as part of its National Program for Happiness and Wellbeing initiative, which included voluntary traffic light labeling for fat, saturated fat, sugars, and salt levels on prepackaged foods [28,29]. In contrast to 11.76% of the MNC products, none of the SME labneh products incorporated a traffic light nutrition panel (Figure 2). Both MNCs (29.41%) and SMEs (35%) were comparable in terms of the % labneh products that included vitamins. Similarly, both MNCs and SMEs mirrored each other in terms of % products reporting salt content, with 60% of the products of each source reporting salt content (Figure 2).

Given the consumer preference for local products, the country-of-origin label declaration is of significance [24]. As elaborated previously, in terms of the % products of local origin, UAE was listed as the country of origin in their label declaration for 82% of the SME products, which was much higher than the 11.76% of local products among the MNCs (Figure 2). Furthermore, the consumers’ perception of food product quality is often associated with local production [24]. Considering the differences between products produced locally by MNCs and SMEs, a comparison was undertaken to evaluate if the included ingredients between the MNC and SME products varied. It was found that 21% of the SME products included added spices/herbs flavors as opposed to none from the MNCs. On the other hand, while 11.76% of the MNC labneh products featured unique flavors such as honey and chocolate, no such flavored labneh products were found in any of the SME manufactured products assessed (Figure 2).

The ingredient list is reported to guide consumers in making purchase decisions [30]. Among many factors, health concerns related to food ingredients have driven the shift in consumer preference in flavor of foods considered more natural. The growing consumer demand for natural foods has propelled the clean label movement. Formulating clean label foods has posed challenges for manufacturers, not only due to the high costs of natural ingredients, but also due to their inability to achieve similar formulation characteristics as the highly processed ingredients [31]. A comparison between the SME and MNC labneh products was undertaken to evaluate differences in the percentage of products that included preservatives/stabilizers/processing aids and an extra process step of post-fermentation heat treatment. Interestingly, the % products that had preservatives/stabilizers/processing aids as included ingredients did not vary significantly between MNCs (64.71%) and SMEs (67%). In contrast while 100% of MNC labneh products were post-fermentation heat-treated, only 7% of the SME products were declared as post-fermentation heat-treated (Figure 2). All MNC products were post-fermentation heat-treated whether they originated locally in the UAE or were sourced from overseas manufacturers; hence, the time of their transport to the UAE market is not a case for differentiation (Figure 2). Since all MNC products surveyed were 100% heat-treated, the observations suggest there are no differences due to the style of labneh sold by MNCs (Figure 2). Additionally, 5 out of 28 SME products were imported; however, only 7% of SME labneh were heat-treated. This again seems to indicate that MNCs probably use post-heat treatment as an additional food safety control due to their products being more widely distributed and at a larger scale than their SME counterparts.

### 3.5. The Nutrition Dimension

The nutrition facts panel has been employed by manufacturers as a cost-effective tool to communicate product nutrition information to consumers at the point of purchase. It has not only been proposed as an efficient nutrition education tool but has also suggested to promote healthy eating behaviors, leading to reduced sugar, fat, and energy intake, aligning with WHO’s regional nutrition strategy [32,33].

A variation in nutritional properties of fermented milks due to differences in manufacturing practices and manufacturing locations has been reviewed in the literature [34]. Furthermore, the production method of labneh has been reported to influence its nutritional profile [9]. A significant association of labneh’s brand type with compositional variables has been shown in previous studies [35]. The current study compared the variations in the nutritional composition between the SME and MNC labneh products.

**Figure 3:**
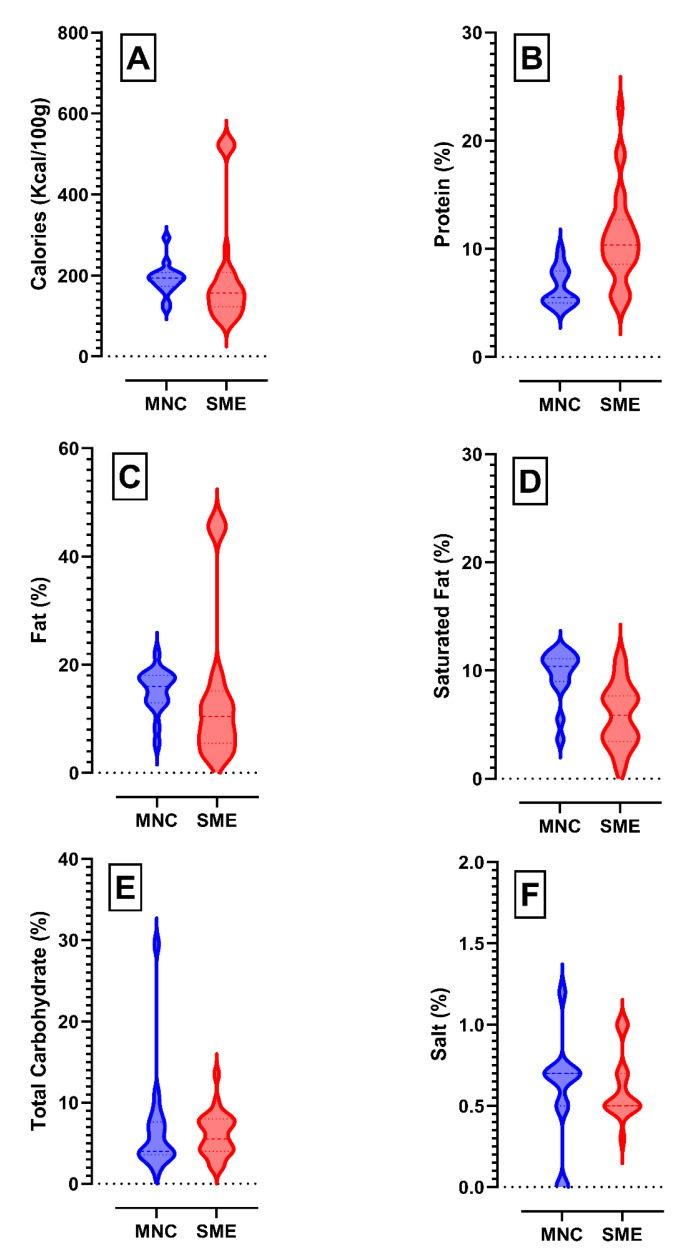
Density plot of reported nutritional content of SME vs. MNC labneh products. (**A**) Calorific content, (**B**) % protein, (**C**) % fat, (**D**) % saturated fat, (**E**) % total carbohydrate, and (**F**) = % salt.

Calories and protein were some of the most frequently reported items in the nutritional facts panel, with these being listed in 100% of both MNC and SME products. The calorific content of the MNC products ranged between 118.2 and 293 Kcal/100 g. The calorific range of 84.5 to 522 Kcal/100 g was observed for SME products (Figure 3A). On the other hand, the range of the reported protein content of the MNC labneh products (4.5–10%; median 5.50%) was significantly higher (*p* < 0.0001) than that of the SME products (5–23%; median 10.35%), as shown in Figure 3B.

Interestingly, 88.2% and 64.7% of the MNC products reported the saturated fat and cholesterol content, respectively, while only 42.9% and 28.6% of the SME products reported these respective values. The reported fat content of MNC labneh products (5.5–22%; median 16.00%) was also significantly higher (*p* < 0.01) than that of the SME products (2.7–45.6%; median 10.45%). The saturated fat content of the MNC products (Figure 3C,D) ranged between 3.6 and 12% (median 10.40%), significantly higher (*p* < 0.001) than that of the SME products (1.2–11.0%; median 5.85%). The reported fat content of the majority of the labneh products surveyed in this study was observed to lie above 3.5% (full-fat), with only two SME products being reported as low-fat.

The carbohydrate content was reported in all the surveyed MNC products with 94% of MNC products also reporting the total sugar content. In contrast, 96% of surveyed SME products reported the carbohydrate content and only 50% of the SME products reported the total sugar content. The carbohydrate content of the MNC products varied widely, ranging between 1.6 and 29.5% (Figure 3E), with the total sugar content varying between 1 and 20%, while the SME product carbohydrate (Figure 3E) and total sugar content ranged from 2.0 to 13.5% and 2.8 to 8.2%, respectively.

Due to the adverse health outcomes linked to excessive salt consumption, the WHO has recommended <5 g per person per day (2 g/d of sodium) for adults [28]. The average salt intake in the UAE was reported to be around 9 g/d (≈3.5 g/d of sodium), which is much higher than the recommended values. Therefore, the nutritional panel reporting of salt content is imperative for assessing the salt intake [27,36]. The salt content of MNC and SME products was similar, with the MNC products ranging between 0 and 1.2% and SME products varying between 0.3 and 1% (Figure 3F). Of the 16 (94.1%) MNC products reporting the sodium content, the value ranged between 0.13 and 0.45%. On the other hand, only 64.3% of the SME products reported the sodium content, and the values ranged from 0.16 to 1.167%.

*Part 2: Physicochemical Properties of Selected Labneh Products*

An in-depth analysis of the physicochemical properties of select locally available labneh was undertaken. The survey of local retail stores identified 35 non-flavored labneh products, which included 18 packaged and 17 loose labneh. Labneh sold loose was excluded from the study due to limited product and compositional information availability, and the 18 packaged labneh were reviewed for differences in formulation, processing, and composition, both from their label and website information.

As elaborated in the previous section, the fat content of the majority of labneh samples available in the UAE market was above 3.5%; however, a wide variation of fat content was observed among them. Therefore, a stratified convenience sampling technique was employed, and available packaged labneh, categorized into three groups, high-fat, full-fat, and lite-fat, were procured for the study. Three products from each category were identified (Table 2).

Another observation as elaborated in the previous section was the practice of including preservatives/stabilizers/processing aids in the ingredients list. Almost 60 to 70% of labneh products surveyed in the UAE market reported the incorporated preservatives/stabilizers/processing aids in their labneh products. The dosage and ratios of individual additives could also play an important role in determining product quality and its physicochemical characteristics. Therefore, to evaluate the influence of these additives on the physicochemical characteristics of labneh, a further selection was employed such that at least one product was without additives. Samples were selected considering their availability at the time of the study. The differences in processing, composition, shelf life, etc., were also noted. When collecting samples, one of the lite-fat labneh was not available in the market; hence, only two lite-fat labneh were evaluated.

As per the manufacturer’s published information, among the high-fat labneh, H1 samples had added sodium alginate and guar gum and were made using only starter cultures. The H2 labneh did not have any gelling or thickening agents added (as per the label declaration) and was made with only starter cultures. The H3 labneh was made with the addition of pectin and polyphosphates. Moreover, H3 labneh was made with a rennet addition along with a starter culture, which was later destroyed with a post-fermentation kill step. All high-fat labneh samples underwent a heat treatment after the fermentation step. The application of post-fermentation heat treatment was potentially the underlying factor contributing to the extended shelf life of labneh H1–H3.

When reviewing the manufacturer’s published information of full-fat labneh, two of them did not have any additives and were made using only starter cultures (without rennet) and were not heat-treated after the fermentation process step. The compositional information of the F1 full-fat labneh revealed the inclusion of non-dairy fat ingredients, carrageenan, starch, and guar gum, and it was coagulated using microbial rennet. The labneh was not heat processed post-fermentation. Since none of the full-fat labneh had heat treatment after fermentation, they all had a reduced shelf life of 14–21 days (Table 2).

The labels and the product information from the website did not indicate any use of additives in the formulation of either lite-fat labneh. However, a difference in the specified shelf life between the two lite-fat labneh L1 and L2 was noted, wherein L2 had a shorter shelf life of 21 d, while L1 was indicted to have a longer shelf life of 120 d, potentially due to post-fermentation processing (although this was not indicated on the label or website).

Strategies such as post-production heat treatments or alternative treatments as permitted under GSO regulation are often employed to extend the shelf life and reduce post-fermentation acidification. Other techniques, such as inclusion of additives and carbonization, etc., are also engaged to extend the product shelf life [11]. The long shelf life of L1 of 120 d was similar to that of other post-fermentation heat-treated labneh, while L2 only had a 21 d shelf life, similar to non-heat-treated labneh.

### 3.6. Proximate Composition

The chemical composition of labneh samples classified as high-fat, full-fat and lite-fat are summarized in Table 3. The labneh differed significantly among the three categories in their fat and protein contents. The high-fat labneh had a 17–18% fat content, while the fat content was halved (7–8%) in the full-fat labneh and again halved (3.5–4.5%) in the lite-fat labneh. F1 labneh was indicated to have added mono- and diglycerides of fatty acids. The protein contents followed an opposite trend such that high-fat labneh had the least levels of protein (4.9–7.5%), followed by full-fat labneh (8.6–12.7%), and the highest (14.7–15%) protein content was in the lite-fat labneh. The carbohydrate contents were not significantly different for the three categories of labneh, given that within each category there was a huge variation leading to an overlap in the range values among the three categories. The composition of full-fat labneh was similar to that of the traditional labneh [37].

### 3.7. Rheological Analysis

In amplitude sweep measurements, the frequency was kept constant at 0.25 Hz while increasing the deflection in a stepwise manner. The limit of viscoelastic region (LVE) was determined at which the test could be carried out without destroying the structure of the samples, i.e., G′ showed a constant value. The tolerance range of ±10% deviation for G′ around the plateau value was selected so that the samples showed LVE behavior. The % strain value was determined in the middle of the LVE. This fixed strain was used to conduct frequency sweep tests, guaranteeing the measurements were within the viscoelastic regime. The tan δ values for all tests were below 1, indicating the predominantly elastic behavior of the samples.

All samples of labneh showed a viscoelastic solid structure similar to a gel (G′ > G″ in LVE) when measured at 0.25 Hz. The curve of the G′ function showed a continuous drop after leaving the LVE region, indicating creamy behavior with a gradual breakdown of the network structure within the labneh samples. Upon a comparison of the flow transition index, the lite-fat labneh (<5% fat) showed less creamy behavior and showed values closer to brittle fracture [38], which confirmed that the tested lite-fat labneh did not contain any gelling or thickening agents, as verified from their ingredient lists (Table 2). However, the flow transition index measures did not differentiate (significantly) samples of labneh containing full- or high-fat.

The consistency of the gel structure and the resistance of the gel network to the applied strain was evaluated by determining the complex viscosity (η*) and complex modulus (G*), respectively [39,40]. In jellified structures, the release of taste and aroma compounds in the mouth is reported to depend on gel disintegration [41]. A correlation of the complex viscosity and complex modulus with sensory aspects such as perceived firmness, thickness, and creaminess has been reported in the literature [42,43,44]. Furthermore, for the same matrix composition, the in-nose release and perception has been reported to be influenced by complex viscosity [42]. The high-fat labneh with a higher total solids content showed a significantly higher complex viscosity and complex modulus compared to the full-fat and lite-fat labneh. The high-fat labneh containing pectin (H3) showed the highest complex viscosity and complex modulus with significantly higher values than H2 and H1. Similar trends were also observed for the complex modulus of the tested high- and lite-fat labneh samples. These findings of our study are supported by the literature, wherein factors such as the proximate composition, moisture content, and presence of hydrocolloid have all been reported to influence the complex viscosity and complex modulus [45]. In contrast to the reported literature, the full-fat labneh (F1) with an added mix of hydrocolloids (starch, guar gum, and carrageenan) had a significantly lower complex modulus, differing from that of the full-fat labneh F2 and F3 despite the presence of hydrocolloids.

**Figure 4:**
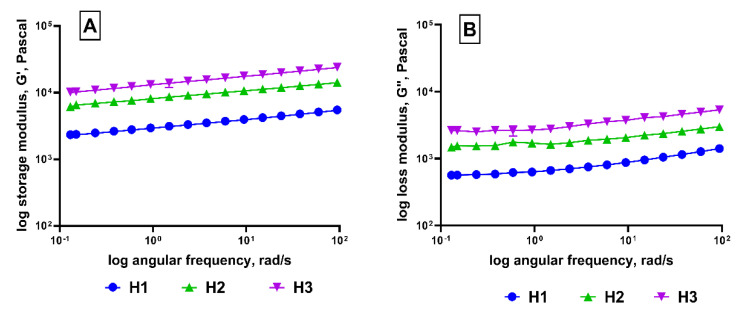
Rheological differences. (**A**) Storage modulus and (**B**) loss modulus measured for high-fat labneh. H1, H2—control, and H3. (**A**) Storage modulus measured over a range of angular frequencies. (**B**) Loss modulus measured using frequency sweep. (Represented data points are Mean ± SEM).

The frequency sweep results of the rheology of the high-fat labneh are shown in Figure 4A. High-fat labneh containing pectin (H3) showed dominant solid-like properties displaying a higher storage modulus and loss modulus when compared to H2 (additive free control). This trend was observed during the two weeks of measurement. The storage modulus indicates the ability of a material to store energy elastically, and the increasing storage modulus suggests that the strength of bonds in the protein structure might have been enhanced with the pectin addition [46]. High methoxyl pectin (HMP) is a preferred hydrocolloid for acidic systems [47], wherein caseins are less stable. Pectin being anionic, protein-pectin bonding results due to its adsorption onto casein micelles via electrostatic interactions at acidic pH [48]. Also, HMP forms calcium linkages with caseins, thus providing increased stability to the caseins. This prevents casein aggregation, sedimentation, and serum separation from the labneh system [49]. However, increasing these stable linkages in a solid gel makes them more rigid and inflexible, and therefore, an enhanced storage modulus and loss modulus were observed at increasing frequencies. These results agree with the literature, wherein inclusion of pectins in emulsions has been reported to increase the storage and loss modulus [45]. In comparison, high-fat (H1) labneh with added alginate and guar gum showed a lower storage modulus (Figure 4A) and loss modulus (Figure 4B), similar to findings in the literature [50,51], indicating its more liquid consistency and weaker gel network. Differences in the rheological behavior based on the inclusion of hydrocolloids and stabilizers in the formulation has been reported for cream cheeses [52].

The differences between H3 and H1 could be attributed to the presence of pectin in the former in contrast to the alginate and guar gum in the latter. Studies reported that the gelation mechanism between alginate and pectin are different and are also affected by many extrinsic factors, such as the pH, presence of co-solutes, temperature, etc. [53]. All the high-fat labneh were heat-treated and hence had long shelf life.

**Figure 5:**
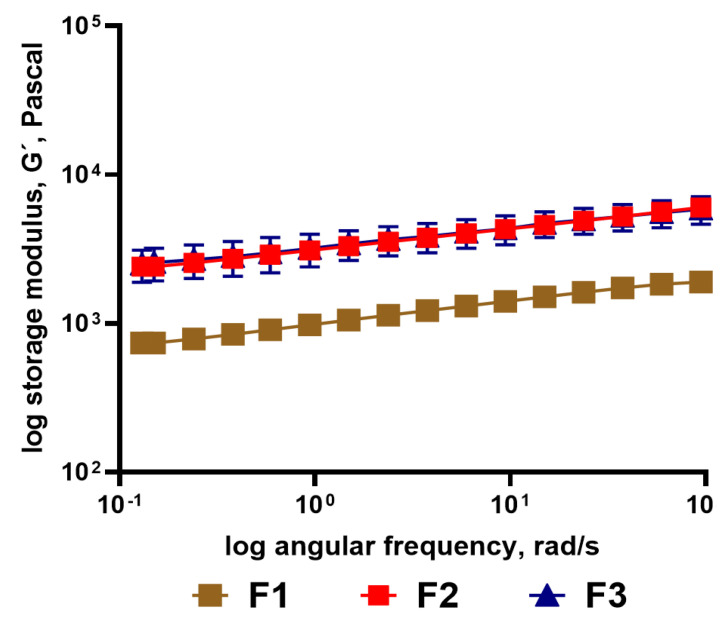
Rheological differences in measured storage modulus during frequency sweep for full-fat (F2/F3 control and F1 with additives) labneh. (Represented data points are Mean ± SEM).

Full-fat labneh F1, with an added mix of hydrocolloids (starch, guar gum, and carrageenan) had a lower storage modulus (Figure 5), indicating weaker structure compared to the control (no hydrocolloid added) full-fat labneh. The loss of deformation energy was similar for all full-fat labneh.

Hydrocolloids are frequently used in the food industry as thickening agents [54]. But, several factors, such as the type of hydrocolloid used and its concentration, the co-solutes present, the type of food system, and the pH and temperature of the food system all influence the thickening effect and the rheological status of hydrocolloid gels [54]. Our findings were also supported by the literature wherein modified starch was reported to impart a large influence on the gel’s complex modulus [55]. None of the full-fat labneh were heat-treated post-fermentation, and hence had 2–3 weeks of shelf life.

**Figure 6:**
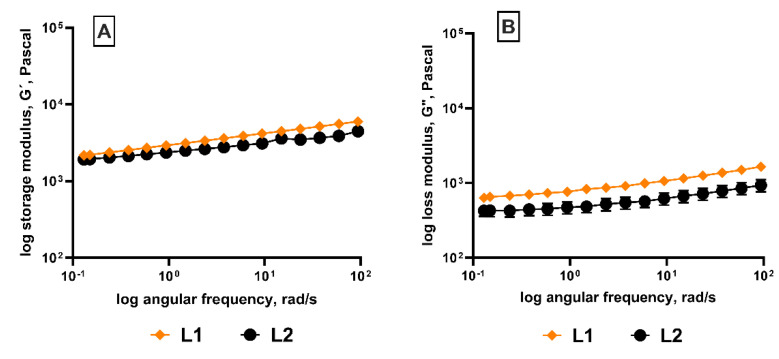
Rheological differences. (**A**) Storage modulus and (**B**) loss modulus measured for lite-fat labneh L1 and L2 (both labneh without additives). (**A**) Storage modulus lite-fat when subjected to oscillation stress. (**B**) Loss modulus of lite-fat labneh measured using frequency sweep. (Represented data points are Mean ± SEM).

The two lite-fat labneh had a similar overall structural stability (Figure 6A,B) when subjected to shear. The storage modulus was similar for heat-treated and non-heat-treated (control) lite-fat labneh. Only the loss modulus and the viscous modulus seemed to differ, with the heat-treated (L1 labneh) showing higher G″ values than the control (L2 labneh). Post-fermentation heat treatment has been reported to alter the viscosity of the product [56]. Furthermore, as previously reported in the literature, the storage modulus (G′) was greater than the loss modulus (G″) over the measured range and appeared to be frequency dependent [23].

### 3.8. Texture Analysis

**Figure 7:**
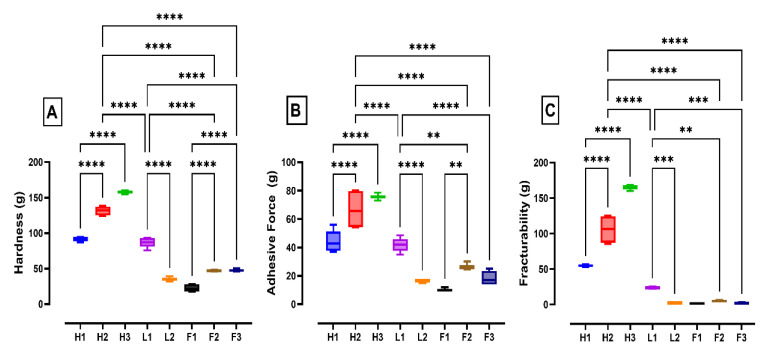
Texture characteristics. (**A**) Hardness, (**B**) adhesiveness, and (**C**) fracturability) of high-fat containing (H1–H3), full-fat containing (F1–F3), and lite-fat containing (L1, L2) labneh. In this figure ** means *p* < 0.01; *** means *p* < 0.001 and **** means *p* < 0.0001. The *p* values indicate the level of significance as per ANOVA analysis.

Hardness, adhesiveness, stringiness, and fracturability were measured for the selected labneh samples. Hardness (g) is the force required to achieve a certain deformation and can be described from a sensory point of view as the power required for compressing a sample between molar teeth [57]. The hardness of the tested labneh samples ranged from 32 g to 160 g (Figure 7A).

As previously reported in the literature, fat levels impacted the hardness values of the tested labneh samples. The hardness values of the high-fat labneh were higher than those of the full/lite-fat labneh and similar to results reported in literature [58,59,60]. The results agreed with the observation that the hardness of commercial cream cheese increased with a high-fat and low-moisture content, producing harder spreadability due to the firm and stiff texture [61]. The differences in textural characteristics were probably due to higher total solids and higher fat content in high-fat labneh [62]. As opposed to the high-fat labneh, the full-fat labneh had the lowest hardness values. The hardness values for lite-fat labneh were between those of the high-fat and full-fat labneh. Similar findings have been reported in literature wherein the hardness of low-fat cheese was more than that of full-fat cheese [63,64,65,66]. The higher hardness values observed in lite-fat labneh can be attributed to the higher calcium amount along with the larger structural protein matrix per unit cross-sectional area for these labneh [67,68].

Among the high-fat labneh, those formulated with pectin (H3) showed significantly greater hardness than H2 (high-fat labneh with no additives) and H1 (labneh with added alginate and guar gum). The literature reports an association between the addition of pectin with changes to the textural profile, such as increased hardness [46,65]. Hydrocolloids such as pectin, which is an anionic polysaccharide, are widely used to stabilize dairy products to improve the final product texture [45]. The improved structural stability due to the addition of pectin is attributed to casein-pectin complexes, wherein the pectin adsorbs to the casein micelle surfaces and, through a combination of electrostatic and steric stabilization, prevent casein aggregation in acidified milk [46,65]. H1 labneh (with alginate and guar gum) had the lowest hardness values as compared to H2 and H3 labneh, possibly due to the presence of guar gum and alginate. While the addition of guar gum has also been associated with improving texture and decreasing hardness values in cheese and yogurt [69,70], the mechanistic basis of such association is poorly understood. Hege et al. [71] showed that the addition of guar gum to milk does not lead to interactions with the caseins and whey proteins, but rather the proteins and the guar gum remain in separate phases. However, alginate is reported to soften the milk protein gel by inducing formation of particles that break the protein network of the gel, due to its high-water-holding capacity [62].

A similar trend was observed in the full-fat labneh and F1 labneh with an added mix of starch, guar gum, and carrageenan, which had significantly lower hardness values compared to other fresh full-fat labneh. Although there are differences in the interactions between the different hydrocolloids with the caseins and whey proteins in milk, the benefits of such interactions should be pronounced if these hydrocolloids are added to the milk system prior to making labneh. Along with the impact of guar gum on the labneh texture, the addition of carrageenan to milk has also been shown to lead to interactions with caseins [72]. In the commercial labneh, the addition of carrageenan, guar gum, starch, and mono/di-glycerides in F1 labneh manufacturing showed a significantly lower hardness and adhesiveness compared to an additive-free (F2) labneh. These full-fat labneh have a low shelf life as they were not heat-treated post-fermentation. Therefore, the differences in the textural and hardness values of labneh samples tested in our study could potentially be attributed to the differences in the incorporation of hydrocolloids and differences in post-fermentation processing.

Other textural characteristics measured in the study included adhesiveness, stringiness, and fracturability. These characteristics of the labneh samples tested also showed similar trends in the hardness values and correlated with their total solids content (Figure 7B,C). Overall, the texture analysis results showed that high-fat labneh were more elastic, harder, and have greater fracturability and adhesiveness, while they were less stringy than full/lite-fat labneh.

### 3.9. Principal Component Analysis

**Figure 8:**
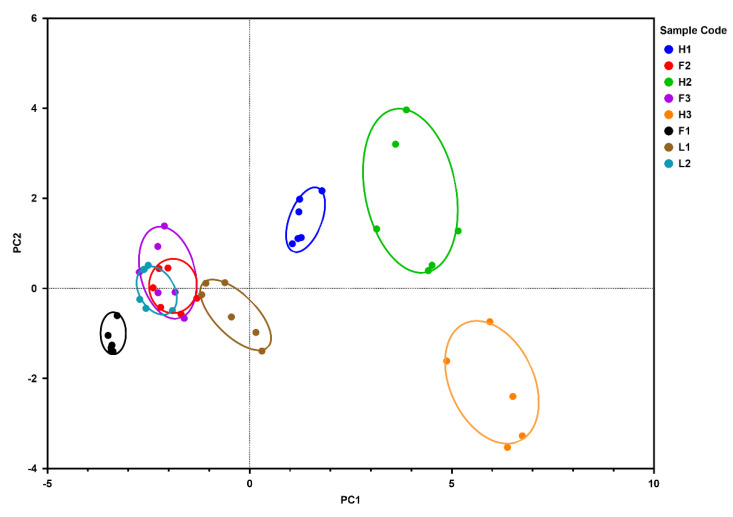
PCA of rheological and texture characteristics by type of labneh showing high-fat containing (H1–H3), full-fat containing (F1–F3), and lite-fat containing (L1, L2) labneh.

PCA is a popular data mining statistical method employed to characterize multidimensional data and to summarize variations in complex data analysis. Principal component analysis (PCA) was applied to the mean values to evaluate the association of the tested labneh samples with the physicochemical and textural properties and the variability of viscoelastic parameters. The two principal components (PCs) explained 71.57% of the variation in the viscoelastic behavior; PC1 (59.51%) and PC2 (12.06%). The samples were clearly separated along the differences in the formulations and fat content of labneh (Figure 8).

Despite the differences in the observed textural and rheological characteristics, clustering within a group of similar fat-containing labneh was noted. This is in line with the previously reported literature wherein separation of “fat related attributes” were noted across one of their principal components [73]. Samples with a high-fat content (H1–H3) were in the positive part of the first dimension, while the full-fat and the lite-fat samples were in the negative part of the first dimension (Figure 8).

The clustering of H1 labneh samples and H2 labneh samples in the first quadrant indicated that H1 and H2 samples had quite similar attributes, with both positively correlating with PC1 and PC2. In contrast, H3 was clustered in the fourth quadrant wherein the rheological parameters of flow transition index, complex modulus, and complex viscosity, along with load at target and hardness positively corelated to PC1, while negatively corelating to PC2.

The lite-fat and full-fat labneh were clustered around the left side quadrants and, despite not including shelf life as a parameter for the multivariate analysis, a separation along the lines of shelf stability was observed (Figure 8). With a shelf life of 120 days, L1 was clustered closer to the H1–H3 clusters, indicating that post-fermentation processing such as heat treatment to improve the labneh shelf life influences the textural and rheological characteristics of the product. Clusters of short shelf life products L2, F3, F2, and F1 were located together, and an overlap between F2, F3 and L2 clusters was observed. The F1 cluster was separated from the others, indicating that the inclusion of hydrocolloid additives such as carrageenan, starch, and guar gum affected the textural and rheological characteristics of the labneh.

**Figure 9:**
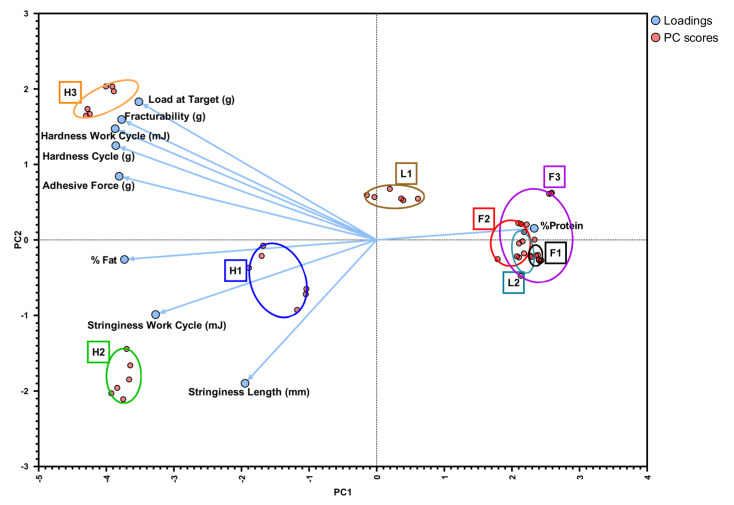
Biplot of textural characteristics by type of Labneh sample of high-fat (H1–H3), full-fat containing (F1–F3), and lite-fat containing (L1, L2) labneh.

To further dissect and evaluate the association of physicochemical parameters of the samples with the textural properties, an additional PCA analysis was performed. The two principal components (PCs) explained 87.61% of the variation in the viscoelastic behavior; PC1 (76.38%), and PC2 (11.24%) and the correlations between composition and textural characteristics were shown to be associated by the type of labneh. In the biplot, H3 labneh made with added pectin, citrates, and polyphosphates were clustered in the second quadrant, wherein the textural characteristics, namely load at target, fracturability, and hardness, negatively corelated to PC1, while positively corelating with PC2. The uniqueness of the texture characteristics of H3 labneh relative to H1 and H2 labnehs could be primarily due to enzymes (microbial rennet), exacerbated by the use of different hydrocolloids in their formulations. The high-fat labneh without any additives (H2) and H1 labneh (made with added sodium alginate and guar gum) were more closely related, with both clustering in the third quadrant, with the fat % and the stringiness values negatively correlating with PC1 and PC2. As observed in the earlier PC analysis, the clustering of labneh was influenced by their fat content and differences in their textural profile due to the inclusion of hydrocolloids. As with the earlier PCA analysis, the full-fat and lite-fat labneh had overlapping clusters closely related to their protein content and texture profile characteristics (Figure 9). The separation of the L1 labneh cluster on the biplot suggests that the additional heat treatment given post-fermentation affected the textural characteristics.

Clustering of the short shelf life labneh was observed towards the right of the biplot, and positively correlated with PC1. All labneh heat-treated post-fermentation showed a shift towards the left of the biplot, indicating that the effects of secondary heat treatment at a low pH could have further altered the protein interactions by incorporating more whey proteins into the network matrix, as reported in the case of cream cheese [74] and in camel milk labneh [75]. In both cream cheese and camel milk labneh, the network structure was more compact when heat treatment was increased.

## 4. Conclusions

A UAE market research analysis of 116 labneh products found that 89.4% of unpackaged labneh, 11.8% of the MNC products, 82.1% of the SME products, and 100% of the specialty products were manufactured locally. The ingredient listing for unpackaged and specialty products was unclear, whereas MNC products listed 3–21 ingredients and SME products listed 3–9 ingredients. About 65% of MNC and SME products contained additives, such as stabilizers, emulsifiers, thickeners, gelling agents, preservatives, processing aids, etc. Specialty labneh products were sold at premium prices relative to MNC and SME products. The MNC products had higher protein and fat contents and more of these products reported saturated fat and cholesterol content in their nutrition table than SME products. The many unpackaged, packaged, and specialty labneh have various product descriptors contributing to their variety, and the complexity was further exacerbated due to variations in style and manufacture process. The labneh was produced by acid coagulation from fermentation or in combination with rennet enzymes and heat-treated as an optional step to increase the shelf life from 21 to 120 days. The study found differences in composition, formulation, instrumental textural characteristics, and rheology among the analyzed commercial labneh. All labneh samples showed viscoelastic solid structures similar to a gel. High-fat labneh with higher total solids showed a higher complex viscosity and complex modulus compared to full-fat and lite-fat labneh. Additives such as starch, alginates, and guar gum in formulated full-fat labneh reduced the complex modulus, storage modulus, loss modulus, and textural characteristics. Furthermore, composition, formulation, and processing-based clustering were observed in PCA analysis as influencing the physicochemical characteristics of the labneh. Further research is needed to examine the impact of formulation design changes on rheological and textural characteristics.

## Figures and Tables

**Table 1:** Packaged Labneh product characteristics in UAE market.

Product Type	Product Description	Style of Labneh Products	Labneh Flavors/Spices	Milk Sourced from Species	Additives Reported	Retailer Sources
**Unpackaged**	“Authentic”, “Fresh”, “Soft”, “Deli Selection”, “Hard”, “Labaneh Jarashieh”, “Full Fat”, and “Low Fat” labneh or labneh ball are also described based on herbs/spices/condiments used	Labneh; labneh ball; labneh analog; Turkish style; Labanese style.	Plain and herbed	Cow	Not Available	Carrefour, Lulu supermarket, Spar supermarket, Spinneys, Choitrams, and Grand Hyper Mart
**MNC and SME**	Lactose-free, Fresh, Cheese Spread with extra labneh taste, Full-fat, Thick and creamy, Full cream, Premium, Organic, Low-fat/light/lite.	Labneh ball, Jarshia, Fresh, Deli Selection, Original (creamy, full-fat, low-fat), Lactose-free, Organic, Turkish style, Hungarian style, Lebanese style.	Plain, Choco, Honey, Baraka, Chilli, Thyme/Zatar, Chilli and oil, Mint.	Goat, Cow.	E401 (Sodium Alginate), E466/469 (Carboxymethyl Cellulose/Enzymatically Hydrolyzed Carboxymethyl cellulose), E339 (Sodium Phosphates, Sodium Monophosphates), E410 (Locust Bean Gum), E341 (Tri-Calcium Phosphate), E330 (Citric Acid), E440 (Pectin), E452 (Sodium Polyphosphates/Sodium Hexametaphosphate), E407 (Carrageenans), E412 (Guar Gum), E202 (Potassium Sorbate), E235 (Natamycin), E402 (Potassium Alginate), E234 (Nisin), E471 (Glycerol Monostearate, Glycerol Di stearate), E417 (Tara Gum), E341(i) (Calcium Monophosphates), E331 (Sodium Citrate), E322 (Sunflower Lecithin).	Lulu, Carrefour, Grand Hyper Mart, Minbaladeh, Al Ain Co-op, Sharjah Co-op, Spar Supermarket, Spinneys, Choitrams, Noon, Desert Cart, Amazon.ae, Gradoise.
**Specialty Products**	Herbed labneh, Plain labana, Labana salad, Plain labana, Spheral-sheep, Spheral, Description based on herb/nondairy ingredient used	Labneh Mix, Herbed Labneh Mix.	Pomegranate, Makdous, Olive Spicy, Black Seeds, Pizza Mixture, Thyme, Cheese Thyme, Palestinian Mixture, Gazan Mixture, Olives, Tomato, Shatta, Herbed, Spheral- sheep, Rooh Al Ittihad, Spheral, Matafi, Plain, Spacetoon/Beans	Cow, Sheep	Not Reported	Lulu, Amazon.ae, Sharjah Co-op

**Table 2:** Summary information on commercial labneh samples.

ID	Labneh Type	Gelling Type Additives	Thickening/Creaming-Type Additives	Starter Culture (SC) or with Enzyme	Post-Fermentation Heat-Treated	Shelf Life	Group
**H1**	High-Fat	Sodium Alginate	Guar Gum	SC only	Yes	120	
**H2**	High-Fat	NS	NS	SC only	Yes	180	Control
**H3**	High-Fat	Pectin	Sod/Cal polyphosphates, citric acid, citrates	SC and Microbial rennet	Yes	180	
**F1**	Full-Fat	Carrageenan	Starch, Guar gum, mono- and diglycerides of fatty acids	SC and Microbial rennet	No	14	
**F2**	Full-Fat	NS	NS	SC only	No	14	Control
**F3**	Full-Fat	NS	NS	SC only	No	21	Control
**L1**	Lite-Fat	NS	NS	SC only	NS	120	Control
**L2**	Lite-Fat	NS	NS	SC only	No	21	

NS: Not specified.

**Table 3:** Summary composition of labneh as per the package label.

Label Assigned	% Fat	% Protein	% Carbohydrates
**H1**	18.2	5.5	3.6
**H2**	17.1	4.9	6.3
**H3**	18	7.5	4
**Range (H1 to H3)**	17–18	4.9–7.5	3.8–6.3
**F1**	8	8.6	3.7
**F2**	8	11	8
**F3**	7.1	12.7	2.3
**Range (F1 to F3)**	7.1–8	8.6–12.7	2.3–8
**L1**	4.5	15	4.1
**L2**	3.5	14.7	4.3
**Range (L1 and L2)**	3.5–4.5	14.7–15	4.1–4.3

## Data Availability

The original contributions presented in the study are included in the article, further inquiries can be directed to the corresponding author.
